# An ovary transcriptome for all maturational stages of the striped bass (*Morone saxatilis*), a highly advanced perciform fish

**DOI:** 10.1186/1756-0500-5-111

**Published:** 2012-02-21

**Authors:** Benjamin J Reading, Robert W Chapman, Jennifer E Schaff, Elizabeth H Scholl, Charles H Opperman, Craig V Sullivan

**Affiliations:** 1 North Carolina State University, Department of Biology, Raleigh, NC, USA; 2 South Carolina Department of Natural Resources, Charleston, SC, USA; 3 North Carolina State University, Genomic Sciences Laboratory, Raleigh, NC, USA; 4 North Carolina State University, Department of Plant Pathology, Raleigh, NC, USA; 5 Department of Biology, North Carolina State University, Room 127 David Clark Laboratories, Raleigh, NC 27695-7617, USA

## Abstract

**Background:**

The striped bass and its relatives (genus *Morone*) are important fisheries and aquaculture species native to estuaries and rivers of the Atlantic coast and Gulf of Mexico in North America. To open avenues of gene expression research on reproduction and breeding of striped bass, we generated a collection of expressed sequence tags (ESTs) from a complementary DNA (cDNA) library representative of their ovarian transcriptome.

**Results:**

Sequences of a total of 230,151 ESTs (51,259,448 bp) were acquired by Roche 454 pyrosequencing of cDNA pooled from ovarian tissues obtained at all stages of oocyte growth, at ovulation (eggs), and during preovulatory atresia. Quality filtering of ESTs allowed assembly of 11,208 high-quality contigs ≥ 100 bp, including 2,984 contigs 500 bp or longer (average length 895 bp). Blastx comparisons revealed 5,482 gene orthologues (E-value < 10^-3^), of which 4,120 (36.7% of total contigs) were annotated with Gene Ontology terms (E-value < 10^-6^). There were 5,726 remaining unknown unique sequences (51.1% of total contigs). All of the high-quality EST sequences are available in the National Center for Biotechnology Information (NCBI) Short Read Archive (GenBank: SRX007394). Informative contigs were considered to be abundant if they were assembled from groups of ESTs comprising ≥ 0.15% of the total short read sequences (≥ 345 reads/contig). Approximately 52.5% of these abundant contigs were predicted to have predominant ovary expression through digital differential display *in silico *comparisons to zebrafish (*Danio rerio*) UniGene orthologues. Over 1,300 Gene Ontology terms from Biological Process classes of Reproduction, Reproductive process, and Developmental process were assigned to this collection of annotated contigs.

**Conclusions:**

This first large reference sequence database available for the ecologically and economically important temperate basses (genus *Morone*) provides a foundation for gene expression studies in these species. The predicted predominance of ovary gene expression and assignment of directly relevant Gene Ontology classes suggests a powerful utility of this dataset for analysis of ovarian gene expression related to fundamental questions of oogenesis. Additionally, a high definition Agilent 60-mer oligo ovary 'UniClone' microarray with 8 × 15,000 probe format has been designed based on this striped bass transcriptome (eArray Group: Striper Group, Design ID: 029004).

## Background

The striped bass and its relatives in the genus *Morone *(the temperate basses) are ecologically and economically important aquaculture and fisheries species native to estuaries and rivers of the Atlantic coast and Gulf of Mexico in North America [1,2]. Although the striped bass and its hybrids have been reared as commercial aquaculture products in the United States since the late 1980s, little genetic information is available for these species in public databases at the National Center for Biotechnology Information (NCBI) or elsewhere, consisting only of microsatellite DNA markers [3,4], the mitochondrial genome (GenBank: HM447585), and a medium density genetic linkage map [5]. A major factor contributing to restricted growth of hybrid striped bass farming nationwide is reproductive dysfunction of female striped bass, resulting in non-viable eggs, embryos, and larvae [6]. These reproductive failures hamper selective breeding efforts required for species domestication and improvement. The exact cause(s) of poor egg quality and embryonic mortality in farmed fishes, however, still remain to be discovered, making appropriate and timely corrective measures difficult to achieve [review: [7,8]].

Functional genomics has emerged as a major research field and gene expression (transcriptomics) and proteomics studies are promising approaches to gain new insights into reproductive molecular biology [7,9-12]. Marked advancement in striped bass reproductive technology based on such "Omic" analyses is, however, currently restricted due to the lack of an available, comprehensive sequence database for this species or for other members of the genus *Morone *that are important in aquaculture (e.g. hybrid striped bass) or as research models (e.g. white perch, *M. americana*). Transcriptome resources are currently available for other commercially important fishes, including rainbow trout (*Oncorhynchus mykiss*) [13-16], coho salmon (*Oncorhynchus kisutch*) [17], tilapia (*Oreochromis mossambicus*) [18], Atlantic halibut (*Hippoglossus hippoglossus*) [19], Senegalese sole (*Solea senegalensis*) [20], Atlantic salmon (*Salmo salar*) [21], and cod (*Gadus morhua*) [22].

The emergence of pyrosequencing and later generation DNA sequencing technologies has made acquisition of significant genomic resources accessible and affordable for non-model organisms [23-25]. Vast numbers of expressed sequence tags (ESTs) can readily be generated using these methods, providing direct evidence of gene transcription, and collections of such EST sequences are presently the most important resources used for transcriptome exploration [26]. Depending on the number of ESTs sequenced, resulting databases can represent a high proportion of the total number of gene transcripts expressed by a given tissue (i.e. transcriptome), making downstream procedures for transcriptome profiling, such as oligo microarray or real-time quantitative reverse transcription PCR, tractable without the need for an entire genome sequence.

When sequencing depth is limited, organ specific EST collections permit more efficient gene expression analyses using 'UniClone' microarrays, which are comprised of probe sequences isolated from a single organ type [27-30]. UniClone arrays represent a larger proportion of a target organ transcriptome and have reduced redundancy when compared to arrays comprised of ESTs derived from several different tissue types. Additionally, to realize the full benefits of proteomic analyses based on mass spectrometry, species-specific ESTs are required, since algorithms used for spectral analyses (e.g. SEQUEST, Proteome Discoverer Software, Thermo Scientific, West Palm Beach, FL) require a homologous reference sequence database. For non-model organisms, sequence information from even closely related species can be insufficient for the accurate identification of peptides, since these algorithms tend to be conservative and heterospecific amino acid substitutions can result in peptide misidentification or an inability to detect orthologues [31].

Therefore, the goal of the present study was to provide an ovary transcriptome database representative of all stages of oogenesis and atresia in striped bass, one that could provide the requisite foundation for functional genomics and proteomics investigations of reproduction and egg quality in this species and that would support similar studies in the other temperate basses.

## Results

**Figure 1:**
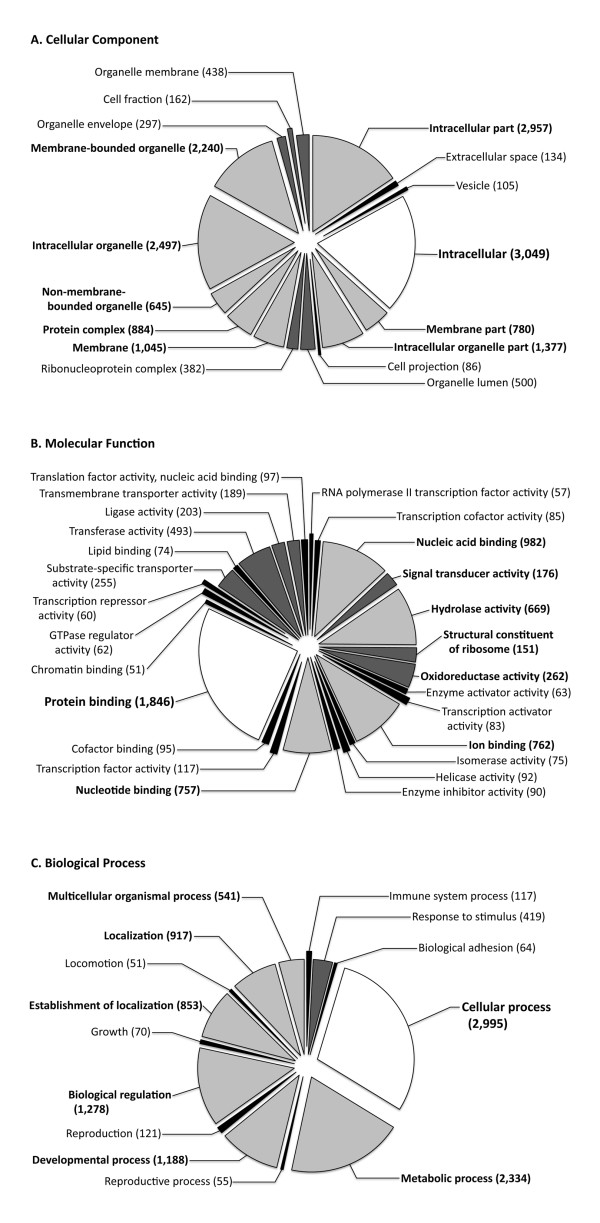
**Gene ontology graph of A**. Cellular Component (3^rd ^level GO terms), B. Molecular Function (3^rd ^level GO terms), and C. Biological Process (2^nd ^level GO terms) of annotated genes in the striped bass ovary transcriptome. The number of GOs in each class is shown and sections that contained 50-150 entities are represented in black, 151-500 by dark gray, 500 and up by light gray, and the predominant class is indicated in white.

A total of 230,151 EST short read sequences with a combined length of 51,259,448 bp (average length 224 bp) were generated from cDNA pooled from ovarian tissues and eggs encompassing the various stages of ovary growth, maturation and atresia. A total of 11,208 high-quality contigs with a length of at least 100 bp were assembled and these included 2,984 contigs that were 500 bp or longer (average length 895 bp; total length 5,068,343 bp) (Additional File 1). Blastx comparisons revealed 5,482 orthologues, of which 4,120 (36.7%) were annotated with Gene Ontology (GO) terms. The number of unknown, unique sequences was 5,726 (51.1%). The breakdown of GO annotation classes within the three categories of GO terms for all annotated sequences is shown in Figure 1: Biological Process (2^nd ^level) and Molecular Function and Cellular Component (3^rd ^level). A complete list, in FASTA format, of the contig assemblies identified by their annotations are included as Additional File 2 and a list of the assemblies and their GO terms are included as Additional File 3.

There were 66 contigs that were each assembled from groups of ESTs that comprised ≥ 0.15% of the total 230,151 reads (i.e. ≥ 345 reads per contig) and these contigs were considered to have abundant ovary expression. These contigs were identified by NCBI UniGene cluster and compared to zebrafish, *Danio rerio*, orthologues evaluated by Digital Differential Display (DDD) (Table 1). Twenty-two striped bass genes from this list (33.3% of the total listed) either had no blastx returns (i.e. were novel), or were identified as being unnamed gene products, or had gene names but no zebrafish UniGene orthologues. These were excluded from further evaluation. Of the remaining informative 44 genes, 23 (52.5%) are predicted to have predominant ovary expression based on DDD of zebrafish orthologues, 11 (25.0%) would be expected to have no difference in expression between ovary and other tissues of the body based on the DDD results, and 10 (22.7%) would likely have predominant expression in other tissues of the body based on the DDD comparison. Overall, the estimated 66 most abundantly expressed striped bass ovary genes were assembled from ~1/6 of the total number of short read sequences (Table 1).

**Table 1:** Transcripts abundantly expressed in the striped bass ovary.

	Contig Number	BLAST 2GO Annotation	Gene	GeneID zebrafish taxid: 7955 orthologue	Assembled contig length (bp)	Number of observe sequence reads	% Total sequence reads (230,151)	Fraction of ESTs that mapped to the zebrafish UniGene by DDD			Zebrafish UniGene
									
								Ovary		Body	
1	10186	cyclin b2	*ccnb2*	368316	1284	1146	0.4979340	0.0025	>	0.0001	Dr.80580

2	10415	zona pellucida glycoprotein	*zp2.3*	114439	1329	1076	0.4675192	0.0429	>	0.0012	Dr.143785

3	10181	novel protein with zona pellucida-like domain	*si: ch211-14a17.7*	368669	646	1001	0.4349318	0.0015	>	0.0001	Dr.75717

4	9349	zona pellucida c	*zpcx*	334011	2036	923	0.4010411	0.0013	>	0.0001	Dr.80433

5	146	nad h quinone 1	*nqo1*	322506	916	908	0.3945236	n.d.	=	n.d.	Dr.4189

6	8878	tubulin beta 2c	*zgc: 123194*	641421	1510	869	0.3775782	n.d.	=	n.d.	Dr.52550

7	9768	egg envelope component zpax	*si: dkeyp-50f7.2*	334036	2890	864	0.3754057	0.0017	>	0.0003	Dr.105787

8	10472	fatty acid binding protein liver	*fabp1b.1*	554095	419	848	0.3684538	n.d.	=	n.d.	Dr.24261

9	9294	--NA--	*--*	--	812	839	0.3645433	--		--	--

10	10137	choriogenin 1	*zp3b*	64692	1389	817	0.3549843	0.0029	>	0.0003	Dr.75734

11	11102	hypothetical protein LOC100049339	*polr2a*	553347	774	767	0.3332595	*		*	Dr.79109

12	11074	--NA--	*--*	--	181	762	0.3310870	--		--	--

13	10663	zgc: 175135 protein	*zgc: 165551*	100003969	636	706	0.3067551	0.0039	>	0.0003	Dr.106137

14	9917	heat shock protein 8	*hspa8*	573376	2266	699	0.3037136	0.0011	<	0.0029	Dr.75087

15	11091	novel protein with zona pellucida-like domain	*LOC100331707*	100331707	1219	675	0.2932857	--		--	--

16	3	--NA--	*--*	--	1585	654	0.2841613	--		--	--

17	11147	fatty acid-binding heart	*fabp11a*	447944	581	638	0.2772093	n.d.	=	n.d.	Dr.78045

18	10883	mgc86501 protein	*wu: ft38e01*	798996	568	623	0.27069919	0.0024	>	0.0002	Dr.106837

19	9329	histone	*h3f3c*	336231	945	619	0.2689539	0.0001	<	0.0003	Dr.75577

20	10302	voltage gated chloride channel domain-containing protein	*--*	--	996	616	0.2676504	--		--	--

21	11112	egg envelope component zpc	*zp3c*	563179	1527	610	0.2650434	0.0002	>	0	Dr.113688

22	30	histone h2a	*LOC573838 (h2af1o)*	100332229	447	607	0.2637399	0.0024	>	0.0002	Dr.75698

23	10079	--NA--	*--*	--	811	585	0.2541810	--		--	--

24	10058	beta-actin	*bactin2*	57935	1874	578	0.2511395	0.0026	<	0.0077	Dr.75125

25	10823	apolipoprotein d	*zgc: 123339*	567972	816	560	0.2433185	*		*	Dr.15815

26	10825	--NA--	*--*	--	154	555	0.2411460	--		--	--

27	10773	hypothetical protein LOC100049339	*--*	30705	756	555	0.2411460	--		--	--

28	6635	h1 histone member oocyte-specific	*h1m*	327403	823	523	0.2272421	n.d.	=	n.d.	Dr.75735

29	11098	adp atp translocase	*slc25a5*	192321	1243	515	0.2237661	0.0015	<	0.0078	Dr.30295

30	127	nucleoside diphosphate kinase b	*nme2b.1*	30083	834	511	0.2220281	n.d.	=	n.d.	Dr.11052

	Contig Number	BLAST 2GO Annotation	Gene	GeneID zebrafish taxid: 7955 Orthologue	Assembled contig length (bp)	Number of observe sequence reads	% Total sequence reads (230,151)	Fraction of ESTs that mapped to the zebrafish UniGene by DDD			Zebrafish UniGene
	
								Ovary		Body	

31	10309	60 s acidic ribosomal protein p0	*rplp0*	58101	932	497	0.2159452	0.0008	<	0.0033	Dr.55617

32	11081	loc494706 protein (oogenesis-related gene)	*org*	100001110	601	495	0.2150762	0.0016	>	0.0001	Dr.80745

33	10120	elongation factor 1 alpha	*efla*	30516	1744	492	0.2137727	0.0032	<	0.0108	Dr.31797

34	10015	heat shock protein 90	*hsp90ab1*	30573	1900	485	0.2107312	0.0006	<	0.0020	Dr.35688

35	11073	unnamed protein product	*--*	--	414	481	0.2089932	--		--	--

36	10797	complement component (3b 4b) receptor 1	*LOC565541*	565541	1696	470	0.2042138	*		*	Dr.91858

37	92	cyclin b1	*ccnb1*	58025	738	470	0.2042138	0.0035	>	0.0002	Dr.121261

38	10403	--NA--	*--*	--	327	469	0.2037793	--		--	--

39	126	karyopherin alpha 2 (rag cohort importin alpha 1)	*zgc: 55877*	406343	1085	469	0.2037793	0.0010	>	0.0002	Dr.20877

40	10948	--NA--	*--*	--	248	465	0.2020413	--		--	--

41	10900	zpb protein	*LOC100334275*	100334275	1561	461	0.2003033	*		*	Dr.141250

42	36	claudin 4	*cldnd*	81583	731	456	0.1981308	0.0004	>	0.0001	Dr.75663

43	216	stathmin 1 oncoprotein 18 variant 8	*stmn1b*	550548	964	450	0.1955238	0	<	0.0004	Dr.105609

44	10949	--NA--	*--*	550134	151	420	0.1824889	--		--	--

45	9337	Securin [Anoplopoma fimbria]	*LOC566690*	566690	435	414	0.1798819	0.0002	>	0	Dr.118007

46	9321	dna replication inhibitor	*gmnn*	368320	1121	412	0.1790129	n.d.	=	n.d.	Dr.119358

47	10986	cell division cycle 20 homolog (cerevisiae)	*cdc20*	406353	1597	410	0.1781439	0.0005	>	0.0001	Dr.105018

48	11071	--NA--	*--*	--	215	402	0.1746679	--		--	--

49	10743	--NA--	*--*	--	273	398	0.1729299	--		--	--

50	1174	cyclin k	*LOC100331304*	100331304	3331	397	0.1724954	0.0009	>	0	Dr.148591

51	10438	ribonucleotide reductase m2 polypeptide	*rrm2*	30733	1621	396	0.1720610	0.0018	>	0.0003	Dr.75098

52	11198	ribosomal protein s20	*rps20*	406485	477	393	0.1707575	0.0014	>	0.0008	Dr.18943

53	11014	karyopherin alpha 2 (rag cohort importin alpha 1)	*kpna2*	436607	534	380	0.1651090	0.0009	>	0.0002	Dr.75709

54	10351	--NA--	*--*	--	299	375	0.1629365	--		--	--

55	10265	unnamed protein product	*--*	--	1075	375	0.1629365	--		--	--

56	771	cytochrome c oxidase copper chaperone	*cox17*	447914	410	375	0.1629365	0.0007	>	0.0001	Dr.82168

57	10107	tubulin, alpha 1c	*MGC171407*	573122	697	374	0.1625020	n.d.	=	n.d.	Dr.120425

58	161	--NA--	*--*	--	2532	371	0.1611985	--		--	--

59	231	epididymal secretory protein e1 precursor	*npc2*	282673	728	360	0.1564190	--		--	--

60	11090	--NA--	*--*	--	308	356	0.1546811	--		--	--

	Contig Number	BLAST 2GO Annotation	Gene	GeneID zebrafish taxid: 7955 orthologue	Assembled contig length (bp)	Number of observed sequence reads	% Total sequence reads (230,151)	Fraction of ESTs that mapped to the zebrafish UniGene by DDD			Zebrafish UniGene
	
								Ovary		Body	

61	10741	ppia protein (pepitidylprolyl isomerase A)	*ppia*	336612	825	356	0.1546811	0.0005	<	0.0011	Dr.104642

62	9354	superoxide dismutase	*sod1*	30553	795	356	0.1546811	n.d.	=	n.d.	Dr.75822

63	10048	ubiquitin b	*ubb*	550134	169	355	0.1542466	n.d.	=	n.d.	Dr. 104259

64	10083	cyclin a2	*ccna2*	192295	2108	351	0.1525086	n.d.	=	n.d.	Dr.121874

65	10746	eukaryotic translation elongation factor 1 gamma	*eef1g*	195822	1533	350	0.1520741	0.0006	<	0.0011	Dr.75657

66	10761	egg envelope component zpax	*si: dkeyp-50f7.2*	334036	2731	347	0.1507706	0.0017	>	0.0003	Dr.105787

				TOTALS	69173	36532	15.8730570				

Genes are ranked (1-66) by number of observed 454 short read sequences used in each contig assembly. Digital Differential Display (DDD) results of orthologous sequences in zebrafish are also shown

Annotation "--NA--"indicates no blastx return; Dashes (--) indicate unknown or data not available; asterisks (*) indicate the UniGene was not present in the EST libraries used for DDD. Sequences with expression differences evaluated by DDD (FET, *P *≤ 0.05) are indicated by ">" (enhanced ovary expression) or "<" (enhanced body expression); "n.d." indicates no significant difference in expression between ovary and body (=)

All of the high-quality ESTs have been deposited in the NCBI Short Read Archive (GenBank: SRX007394) and annotated contigs are posted under "Resources" on the National Animal Genome Research Program Aquaculture Genome Projects website (http://www.animalgenome.org/aquaculture/database/) [32]. These contigs also have been submitted to Agilent Technologies eArray (Santa Clara, CA) for ovary UniClone microarray design (http://www.chem.agilent.com/). We designed a high definition 60-mer SurePrint oligo array with 8 × 15,000 probe format comprised of 11,145 UniGene probes from the transcriptome, plus an additional 3,854 probes printed in duplicate or selected from *Morone *cDNAs available from NCBI or from our own unpublished results (B.J. Reading and C.V. Sullivan, *unpublished data*) and datasets (eArray Group: Striper Group, Design ID: 029004).

## Discussion

This collection of ESTs represents the first contribution of a large reference sequence database for species of the genus *Morone *and provides a basis for future gene expression studies in these temperate basses. Availability of characterized ovarian transcriptomes from fishes other than zebrafish is limited. Partial transcriptomes have been reported for tilapia (474 EST assemblies) [18] and for cod (1,361 EST assemblies) [22]. Several thousand ovarian ESTs have been reported for salmonid fishes [[13,15,33] and references therein], but to our knowledge these have not been assembled into a comprehensive ovarian transcriptome. Numbers of total ESTs currently available in the NCBI EST database for some other commercially important finfishes are as follows: rainbow trout (287,967), coho salmon (4,942), tilapia (Genus *Oreochromis*, 121,346), Atlantic halibut (20,836), Senegalese sole (10,631), Atlantic salmon (498,212), and cod (229,094). Therefore, the 230,151 ESTs reported herein represent a comparatively valuable transcriptome resource for striped bass.

If the 11,208 contigs are considered to be UniGenes, this represents a substantial proportion of the estimated total protein-coding gene transcripts expressed by the striped bass ovary (i.e. transcriptome) as the average number of mRNA transcripts expressed by a single tissue type is estimated to be between 10,000-15,000 [34], but can be as low as 8,200 [35]. Since over 1,300 GOs from Biological Process classes of Reproduction (121), Reproductive process (55), and Developmental process (1,188) were assigned to the annotated contigs (Figure 1), this sequence collection should prove to be a powerful tool for analysis of ovarian gene expression related to fundamental questions of oogenesis.

Approximately 52.5% of the informative contigs considered to have abundant ovary expression (i.e. those with ≥ 345 reads per contig) were also predicted to have predominant expression in striped bass ovary through DDD comparisons to zebrafish orthologues (Table 1). These include cyclin B2 (*ccnb2*, contig10186), several egg envelope and zona pellucida proteins, histone H2A (*h2af1o*, contig00030), oogenesis-related gene (*org*, contig11081), cyclin B1 (*ccnb1 *contig00092), karyopherin alpha 2 (*kpna2*, contigs 00126 and 11014), claudin 4 (*cldnd*, contig00036), securin (*LOC566690*, contig 09337), cell division cycle 20 homolog (*cdc20*, contig10986), cyclin K (*LOC100331304*, contig11174), ribonucleotide reductase M2 polypeptide (*rrm2*, contig10438), ribosomal protein S20 (*rps20*, contig11198), cytochrome C oxidase copper chaperone (*cox17*, contig00771), and epididymal secretory protein E1 (*npc2*, contig00231). Many of these are well-characterized ovary transcripts and several recent and informative papers have been published detailing the functions of these genes and their protein products in fish oocytes and embryos [see: [7,8,13-20,27,28,36-38]]; others are briefly detailed below.

The remaining 47.5% of abundant striped bass ovary genes that were compared to zebrafish orthologues in the DDD were predicted to have indifferent or predominant expression levels in other tissues of the body relative to the ovary. These may represent constitutively expressed genes or those expressed at high levels in the ovary albeit comparatively lower than in other tissues of the body, respectively. Examples of potential genes with constitutive expression include NADH quinone 1 (*nqo1*, contig00146), tubulin (*zgc:123194*, contig08878 and *MGC171407*, contig10107), fatty acid binding proteins (*fabp1b*, contig10472 and *fabp11a*, contig11147), H1 histone member oocyte-specific (*h1m*, contig06635), nucleoside diphosphate kinase B (*nme2b*, contig00127), geminin DNA replication inhibitor (*gmnn*, contig09321), superoxide dismutase (*sod1*, contig09354), ubiquitin B (*ubb*, contig10048), and cyclin A2 (*ccna2*, contig10083). Of these, fatty acid-binding protein heart (*fabp11a*) has been shown to be up-regulated in ovary of rainbow trout females that mature precociously [13] and an orthologue of *h1m *(*H1foo*) is generally considered to be an oocyte specific histone in mouse (*Mus musculus*) [39,40], contrary to the DDD prediction. The UniGene EST Profile of zebrafish *h1m *(Dr. 75735) indicates that it is predominantly expressed in skin, however the second most abundant site of expression is the reproductive system.

The following genes expressed in striped bass ovary are also expressed in zebrafish ovary, however the DDD indicates that they are predominantly expressed in other tissues of the body (Table 1): histone (*h3f3c*, contig09329), beta-actin (*bactin2*, contig10058), ADP/ATP translocase (*slc25a5*, contig11098), 60S acidic ribosomal protein P0 (*rplp0*, contig10309), elongation factor 1 alpha (*ef1a*, contig10120), peptidylprolyl isomerase A (*ppia*, contig10741), eukaryotic translation elongation factor 1 gamma (*eef1g*, contig10746), stathmin 1 oncoprotein 18 variant 8 (*stmn1b*, contig00216), and heat-shock proteins 8 (*hspa8*, contig09917) and 90 (*hsp90ab1*, contig10015). Ovarian representation of gene transcripts that show predominant expression in other tissues of the body is not surprising given the heterogeneous complexity of the ovary, which is comprised of vasculature, blood and other connective tissues, the somatic follicle, and germ cells. Furthermore, most of these genes, for example *ef1a *and *bactin2*, are considered to have constitutively high expression in most tissues, and this is supported by the corresponding zebrafish UniGene EST Profiles (Dr. 31797 and Dr.75125, respectively). There were, however, three exceptional genes whose expression, although considered to be lower in comparison to other tissues of the body by DDD, have been shown to be highly expressed in ovary. Stathmin (*stmn*) is expressed in oocytes and pre-implantation embryos of mice [41] and in cod ovary [22], and Stmn proteins have been detected in zebrafish ovary [36]. Contig00216 encodes a full-length, 147 amino acid Stmn and has been putatively identified as *stmn1b*, however it is highly similar to two zebrafish *stmn *isoforms (95% and 94% amino acid identity with *stmn1b *and *stmn1a*, respectively). Although *stmn1b *has body predominant expression in zebrafish by DDD (Table 1), zebrafish *stmn1a *(UniGene Dr.52664) shows ovary predominant expression and, therefore, contig00216 may actually be orthologous to *stmn1a*. Given the high similarity of this sequence to both zebrafish *stmn1 *isoforms, it is not possible to definitively assign identity without comparison to the other striped bass *stmn *isoform, which is unavailable. Recently, *hsp8 *and *hsp90 *(corresponding to striped bass *hspa8 *and *hsp90ab1*, respectively) have been characterized as some of the most abundant genes expressed in mouse and fish eggs at both the transcript and protein levels [36,37,42].

This inconsistent result may relate to the inherent weaknesses of DDD, since only highly expressed genes are adequately represented in the EST libraries used to conduct the *in silico *comparisons and the Fisher's exact test (FET) is conservative [43]. Although this method does not offer quantitation, ranking of the striped bass contigs by number of short reads used in assembly paired with comparisons to zebrafish orthologues evaluated by DDD proved to be a useful tool for estimating relative ovarian abundance of the striped bass gene transcripts. Reservation must be taken when considering such interspecific DDD comparisons for the purpose of excluding genes that are predicted to have less predominant expression in one tissue compared to another, since they may be highly expressed in both. This is a promising approach for characterization of novel gene transcripts from EST libraries and has recently been used to identify ovary specific genes in zebrafish [44] and rainbow trout [15], however such results should be further validated using an experimental evaluation of gene expression.

The growing oocyte is considered to be largely transcriptionally inactive, acting as a storehouse of specific maternal RNAs, proteins, and other molecules required for competency for fertilization, initiation of zygotic development, and transition to embryonic gene expression [review: [37,38]]. These maternal factors may be stored in oocytes for extended periods of time until use (e.g. months to years). Therefore, a system of regulatory proteins and RNAs must mediate the oocyte cell cycle during growth, ovarian maturation (OM), and zygotic development from fertilization until activation of the embryonic genome at the mid-blastula transition [45]. A number of known cell-cycle regulators and proteins critical for these processes have been identified as predominantly expressed in striped bass ovary (Table 1). Examples include cyclins B1 and B2 (*ccnb1*, *ccnb2*) [46-49], cyclin K (*ccnk*) [50], securin [51], *cdc20 *[27], *kpna2 *[22,52], *gmnn *[53], *h2af1o *[54] and *org *[44]. Transcripts encoding several different cell division and cell cycle regulatory proteins were similarly reported in the ovaries of cod [22] and rainbow trout [13].

Solute carrier protein (SLC) family members are selected to illustrate representation of sequences in the striped bass ovary transcriptome encoding proteins from a large gene series. The SLCs are a diverse group of eukaryotic membrane proteins that control cellular influx and efflux of solutes, including ions, fatty acids, amino acids, sugars, drugs, and vitamins [55,56]. The Human Genome Gene Nomenclature Committee [57] classifies approximately 400 different human SLCs into 47 families. At least one representative protein from 19 (~40.4%) of these families was identified in the striped bass ovary transcriptome (Table 2). Characterization of SLC gene expression in growing oocytes and during OM would be of direct importance to understanding mechanisms of oogenesis and egg quality in light of what is known of oocyte and egg physiology. Due to osmoregulatory requirements imposed by both fresh and marine waters, embryos of egg-laying fishes develop within the confines of an established chorion that becomes osmotically closed after fertilization. Therefore, ovulated eggs must contain all of the water required during embryogenesis as a medium and substrate for biochemical reactions and as a diluent for waste products (e.g. ammonia). Furthermore, water contributes to appropriate egg buoyancy, especially in marine fishes that spawn pelagic eggs. Prior to ovulation, a hyperosmotic solute concentration develops within the oocytes of these species, followed by passive influx of water through aquaporin membrane channels [review: [58,59]]. Inorganic ions have primarily been implicated in this phenomenon, however the exact mechanisms of their entry have not been verified. Bobe et al. [14] demonstrated up regulation of *slc26 *(Pendrin) and *aqp4 *(aquaporin 4) expression in ovary of rainbow trout during OM. Gene transcripts encoding a slc26a6-like protein, along with several other ion transporters (Table 2) and aquaporin 1 (contig08717) were identified in striped bass ovary. This indicates the potential for discovery of previously unknown mechanisms of teleost oocyte hydration by gene expression analyses of these particular SLCs and water transport genes in the striped bass and related species (genus *Morone*), which can tolerate a wide range of environmental salinities.

**Table 2:** Solute carrier family members identified in the striped bass ovary transcriptome

Contig	Gene	Gene ID *Danio *orthologue	Contig Length (bp)	Solute carrier family function
04292^a^	*slc3a2*	796322	629	Heavy subunit of the heteromeric amino acid transporters (Na^+^-independent, transport of large neutral amino acids: phenylalanine, tyrosine, leucine, arginine and tryptophan)

10145	*slc3a2*-like	100003805	1740	Heavy subunit of the heteromeric amino acid transporters (Na^+^-independent, transport of large neutral amino acids: phenylalanine, tyrosine, leucine, arginine and tryptophan)

09132^b^	*slc4a7*	568872	563	Electroneutral Na^+ ^and HCO_3_^-^-dependent cotransporter

11036^c^	*slc7a2*	100007793	815	Cationic amino acid transporter/glycoprotein-associated amino-acid transporter (transport of the cationic amino acids including arginine, lysine and ornithine)

00672^d^	*slc7a8*	100007704	987	Na^+^-independent, transporter of small and large neutral amino acids such as alanine, serine, threonine, cysteine, phenylalanine, tyrosine, leucine, arginine and tryptophan; when associated with Slc3a2, acts as an amino acid exchanger

05979	*slc7a10*	567420	240	Na^+^-independent, high affinity transport of small neutral D- and L-amino acids

04450	*slc9a3r1*	327272	385	Na^+^/H^+ ^exchanger

02807	*slc10a3*	406519	692	Na^+^/bile acid cotransporter

06556	*slc10a4*	556491	249	Na^+^/bile acid cotransporter

03289	*slc12a5*-like	572215	251	Electroneutral cation/Cl^- ^cotransporter (K^+^/Cl^- ^transporter)

04100	*slc19a2*-like	100329244	778	Thiamine transporter

00585	*slc20a1a*	406458	2129	Na^+^-dependent PO_4_^3- ^transporter

05003	*slc20a1b*	321541	246	Na^+^-dependent PO_4_^3- ^transporter

00176	*slc25a3*	322362	1448	Mitochondrial carrier (PO_4_^3- ^transporter)

01147	*slc25a5*	192321	1302	Mitochondrial carrier (ADT/ATP translocator)

01400^e^	*slc25a12*	337675	693	Mitochondrial carrier (aspartate/glutamate transporter)

01037	*slc25a26*	560478	349	Mitochondrial carrier (S-adenosylmethionine transporter)

09234	*slc25a29*	569608	579	Mitochondrial carrier (carnitine/acylcarnitine transporter)

06849	*slc25a43*	796731	254	Mitochondrial carrier

07197^f^	*slc25a46*	436831	251	Mitochondrial carrier

08784	*slc26a6*-like	557779	215	Multifunctional anion exchanger (Pendrin-like; Cl^-^, oxalate, SO_4_^2-^, and HCO_3_^- ^transporter)

04105	*slc27a1*	541410	265	Fatty acid transporter (FATP-1; long-chain fatty acid translocator)

01322^g^	*slc29a1*	563580	260	Facilitative nucleoside transporter (cellular uptake of nucleosides)

05237	*slc30a2*	563540	293	Zinc transporter

06016	*slc30a2*-like	560642	608	Zinc transporter

05293^h^	*slc30a5*	436594	506	Zinc transporter

03716	*slc30a7*	327439	392	Zinc transporter (zinc efflux transporter)

09883	*slc31a2*	--	2142	Copper transporter (low affinity copper uptake)

02632	*slc35a2*	368487	186	Nucleoside-sugar transporter (UDP-galactose transporter)

07709	*slc35e1*-like	100332364	249	Nucleoside-sugar transporter

04693	*slc38a8*-like	795255	414	Na^+^-coupled neutral amino acid transporter

05870	*slc38a9*	562137	243	Na^+^-coupled neutral amino acid transporter

02706	*slc38a11*	550337	347	Na^+^-coupled neutral amino acid transporter

02072	*slc39a3*	321324	414	Metal ion transporter (zinc influx transporter)

08253^i^	*slc39a13*	368686	239	Metal ion transporter (zinc influx transporter)

05275^j^	*slc44a1*	100333377	256	Choline transporter

02670	*slc44a2*-like	321056	269	Choline transporter

07718	*slc44a4*-like	393385	255	Choline transporter

05152	*slc48a1a*	436697	853	Heme transporter hrg1-B

For contigs with superscripted letters, see also the following corresponding contigs: ^a^02586 (*slc3a2*); ^b^07936, ^b^03956 (*slc4a7*); ^c^00241, ^c^09351, ^c^04452 (*slc7a2*); ^d^04260 (*slc7a8*); ^e^07750 (*slc25a12*); ^f^06248 (*slc25a46*); ^g^04062 (*slc29a1*); ^h^09154 (*slc30a5*); ^i^06486 (*slc39a13*); ^j^02053 (*slc44a1*)

## Conclusions

In summary, as we continue to advance our understanding of reproduction in temperate basses of the genus *Morone*, this reference sequence database of ovarian transcripts will provide the requisite foundation for gene expression studies and will open avenues of research related to reproduction and egg quality. Several important candidate genes have already been identified for future study. Furthermore, these sequences have been used to design an ovary UniClone oligo microarray for assessing changes in gene expression during oogenesis and in female striped bass spawning good and poor quality eggs. Our recent deployment of this microarray in a study of striped bass egg quality has allowed us to detect differences in ovarian gene expression explaining and predicting most of the eventual variance in early embryo mortality among good and poor quality spawners.

## Methods

### Sample collection and preparation

Striped bass were reared in outdoor tanks at the N.C. State University Pamlico Aquaculture Field Laboratory [60]. As the striped bass is a group synchronous, single clutch, iteroparous spawner, ovarian tissues were collected by dissection or through ovarian biopsy [61] from females whose most advanced clutch of oocytes/eggs represented one of several stages (≥ 3 females/stage) of oocyte growth (early primary growth oocytes, diameter 49-81 μm; late primary growth oocytes showing evidence of lipid droplet accumulation, diameter 162-184 μm; vitellogenic growth oocytes, diameter 558-764 μm [see:[62][63]]), oocyte maturation (post-vitellogenic and maturing oocytes, diameter > 900 μm), and atresia [64], and ovulated eggs. All samples were preserved in RNA*later*^® ^(Applied Biosystems/Ambion; Austin, TX). Tissues were pooled in equal weight by oocyte/egg stage and total RNA was extracted in TRIzol^® ^Reagent (Invitrogen; Carlsbad, CA). RNA quality was assessed by agarose gel electrophoresis and NanoDrop™ spectrophotometry (Fisher Scientific; Pittsburgh, PA). Dynabeads^® ^(Invitrogen) were used to purify mRNA as described by the manufacturer.

### cDNA library construction and sequencing

Ovary mRNA was submitted for cDNA synthesis at the N.C. State University Genomic Sciences Laboratory (Raleigh, NC). First and second strand cDNA was synthesized from 2.5 μg of Dnase treated mRNA using the SuperScript™ Double-Stranded cDNA Synthesis Kit (Invitrogen) and oligo (dT)_17 _according to the manufacturer. Approximately 2 μg of cDNA was prepared for FLX sequencing using standard Roche protocols [65]. Briefly, cDNA was nebulized to generate fragments averaging ~500 bp in length, fragment ends were repaired, and adapters containing PCR and sequencing primer annealing sites were ligated. Fragments were immobilized on beads, clonally amplified and then sequenced on a 1/2 plate using standard FLX platform (Roche; Indianapolis, IN).

### Sequence assembly and annotation

Short reads were assembled into contigs using Roche's Newbler software (gsAssembler) with default settings except that the minimum overlap was set to 30 bp. Parameters were set to generate files for large contigs (> 500 bp) and for all contigs > 100 bp. High quality contig assemblies were subjected to BLAST (blastx) [66] of the NCBI database and annotated according to the Gene Ontology Consortium [67] using Blast2GO 2048 M version 12.2.0 [10,68,69]. Parameters for blastx were: Expect value 1.0E^-3 ^and HSP Length Cutoff 33. Parameters for the GO annotations were: E-value-hit-filter 1.0E^-6^, Annotation Cutoff 55, GO Weight 5, and HSP-Hit Coverage Cutoff 0. Combined GO graphs for the annotated sequences (4,120 total) were created using percentages of 2^nd ^level GO terms for Biological Process and 3^rd ^level GO terms for Molecular Function and Cellular Component. Represented GO classes were restricted to those with 50 or more entities (sequence cutoff = 50.0); Sequence Filter = 50, Score alpha = 0.6, Node Score Filter = 10. Parameters for the Combined Graphs, Level Pie Configuration were: Ontology Level = Level 2 or 3 as described above.

### Estimation of abundant gene transcripts

Contigs that were assembled from a number of ESTs comprising ≥ 0.15% of the total 230,151 short reads (i.e. those having ≥ 345 reads per contig) were considered to be abundant [see: [38]]. These contigs were ranked by relative abundance and compared to zebrafish orthologues shown to be ovary predominant by NCBI UniGene DDD [70], see: [15,44]. Zebrafish EST libraries were used to determine relative representation by DDD of orthologous UniGene clusters in ovary (104, 986 ESTs; Lib.IDs 20503, 15519, 20772, 20502, 19214, 15930, 9874, 9767) and body tissues excluding gonads (714, 604 ESTs; Lib.IDs 1520, 1521, 15438, 1028, 17704, 17768, 19753, 1522, 19745, 19746, 20694, 20725, 15518, 21372, 19747, 19748, 4913, 9766, 21371, 19741, 19749, 20771, 19739, 19740, 10504, 19737, 13027, 1029, 17276, 15077, 19752, 15517, 2387, 17282, 17284, 19738, 9968, 9993, 14182, 14249, 19217, 24670, 20072, 20071, 19253, 19219, 19218, 19215, 17283, 17275, 14410, 14409, 13866, 12106, 9706, 4264, 1727). Libraries with sequences derived from embryos, larvae, or whole bodies including gonads were excluded. The Fisher's exact test (FET) was used to determine difference between the number of times sequences from the ovary or body libraries were assigned to a specific UniGene cluster (*P *≤ 0.05). Numerical DDD scores of genes with significantly different expression profiles were reported as the fraction of sequences within the EST libraries that mapped to the UniGene cluster.

### Availability of supporting data

The data sets supporting the results of this article are available in the National Center for Biotechnology Information repository, Short Read Archive: SRX007394 and the National Animal Genome Research Program Aquaculture Genome Projects repository, http://www.animalgenome.org/aquaculture/database/.

## Competing interests

The authors declare that they have no competing interests.

## Authors' contributions

BJR conducted the sample preparation, DDD statistical analyses, and drafted the manuscript. JES performed the FLX pyrosequencing and contig sequence assemblies. RWC performed the GO annotations. RWC, EHS, and CHO participated in design of the study and critical review of the manuscript. CVS conceived the study, participated in its design and coordination, and helped draft the manuscript. All authors read and approved the final manuscript.

## Supplementary Material

Additional file 1 **Striped bass ovary contig assemblies in FASTA format**. Click here for file

Additional file 2 **Striped bass ovary contig assemblies identified by their annotations in FASTA format**. Click here for file

Additional file 3 **List of striped bass ovary contig assemblies and their GO terms**. Click here for file
